# Synergistic interactions of nanoparticles and plant growth promoting rhizobacteria enhancing soil-plant systems: a multigenerational perspective

**DOI:** 10.3389/fpls.2024.1376214

**Published:** 2024-04-29

**Authors:** Krishan K. Verma, Abhishek Joshi, Xiu-Peng Song, Shraddha Singh, Aradhna Kumari, Jaya Arora, Santosh Kumar Singh, Manoj Kumar Solanki, Chandra Shekhar Seth, Yang-Rui Li

**Affiliations:** ^1^ Sugarcane Research Institute, Guangxi Academy of Agricultural Sciences/Key Laboratory of Sugarcane Biotechnology and Genetic Improvement (Guangxi), Ministry of Agriculture and Rural Affairs/Guangxi Key Laboratory of Sugarcane Genetic Improvement, Nanning, Guangxi, China; ^2^ Department of Botany, Mohanlal Sukhadia University, Udaipur, Rajasthan, India; ^3^ Nuclear Agriculture and Biotechnology Division, Bhabha Atomic Research Centre, Mumbai, MH, India; ^4^ Homi Bhabha National Institute, Mumbai, MH, India; ^5^ College of Agriculture, Jawaharlal Nehru Krishi Vishwa Vidyalaya, Ganj Basoda, Vidisha, Madhya Pradesh, India; ^6^ Dr. Rajendra Prasad Central Agricultural University, Pusa, Samastipur, Bihar, India; ^7^ Department of Life Sciences and Biological Sciences, IES University, Bhopal, Madhya Pradesh, India; ^8^ Plant Cytogenetics and Molecular Biology Group, Faculty of Natural Sciences, Institute of Biology, Biotechnology and Environmental Protection, University of Silesia in Katowice, Katowice, Poland; ^9^ Department of Botany, University of Delhi, Delhi, India

**Keywords:** agro-ecological responses, food security, plant-microbiome, soil amendment, NPs, PGPR

## Abstract

Sustainable food security and safety are major concerns on a global scale, especially in developed nations. Adverse agroclimatic conditions affect the largest agricultural-producing areas, which reduces the production of crops. Achieving sustainable food safety is challenging because of several factors, such as soil flooding/waterlogging, ultraviolet (UV) rays, acidic/sodic soil, hazardous ions, low and high temperatures, and nutritional imbalances. Plant growth-promoting rhizobacteria (PGPR) are widely employed in *in-vitro* conditions because they are widely recognized as a more environmentally and sustainably friendly approach to increasing crop yield in contaminated and fertile soil. Conversely, the use of nanoparticles (NPs) as an amendment in the soil has recently been proposed as an economical way to enhance the texture of the soil and improving agricultural yields. Nowadays, various research experiments have combined or individually applied with the PGPR and NPs for balancing soil elements and crop yield in response to control and adverse situations, with the expectation that both additives might perform well together. According to several research findings, interactive applications significantly increase sustainable crop yields more than PGPR or NPs alone. The present review summarized the functional and mechanistic basis of the interactive role of PGPR and NPs. However, this article focused on the potential of the research direction to realize the possible interaction of PGPR and NPs at a large scale in the upcoming years.

## Introduction

Recently, soil amendments have been implemented in agroecosystems to promote plant growth and development, especially by adding organic and inorganic nutrients. Adding specific substances to the soil enhances its capacity to sustain plant life (Urra et al., 2019; Rubin et al., 2023; Kumari et al., 2023b). Plant growth-promoting rhizobacteria (PGPR) and nanomaterials (NMs) or nanoparticles (NPs) are under consideration as a novel approach to boost crop productivity and soil fertility (El-Ramady et al., 2022; Upadhayay et al., 2023; Rajput et al., 2023a; Kumari et al., 2024). Several research studies have demonstrated numerous benefits that soil organic amendments may provide, including better soil texture, higher soil fertility, restored soil health, and, ultimately, higher crop yield (Urra et al., 2019; Rani et al., 2023).

Because of the serious risk factors to human health caused by antibiotic-resistant bacteria, antibiotic residues, and antibiotic-resistant genes that exist in agricultural organic amendments, such contaminants in emerging pollutants are still a major problem (Upadhayay et al., 2023). Soil supplements should have attributes including ecological safeguards and negatively affect the fertility of the soil, soil composition, or the enviro-ecosystem (Garbowski et al., 2022; Rajput et al., 2023a). Owing to their unique properties, PGPRs, and NPs have attracted more interest in recent years as potential soil amendments that can mitigate the risk associated with other soil additions under normal and adverse conditions (Chen et al., 2021; Tolisano and Del Buono, 2023; Kumari et al., 2024).

**Figure 1:**
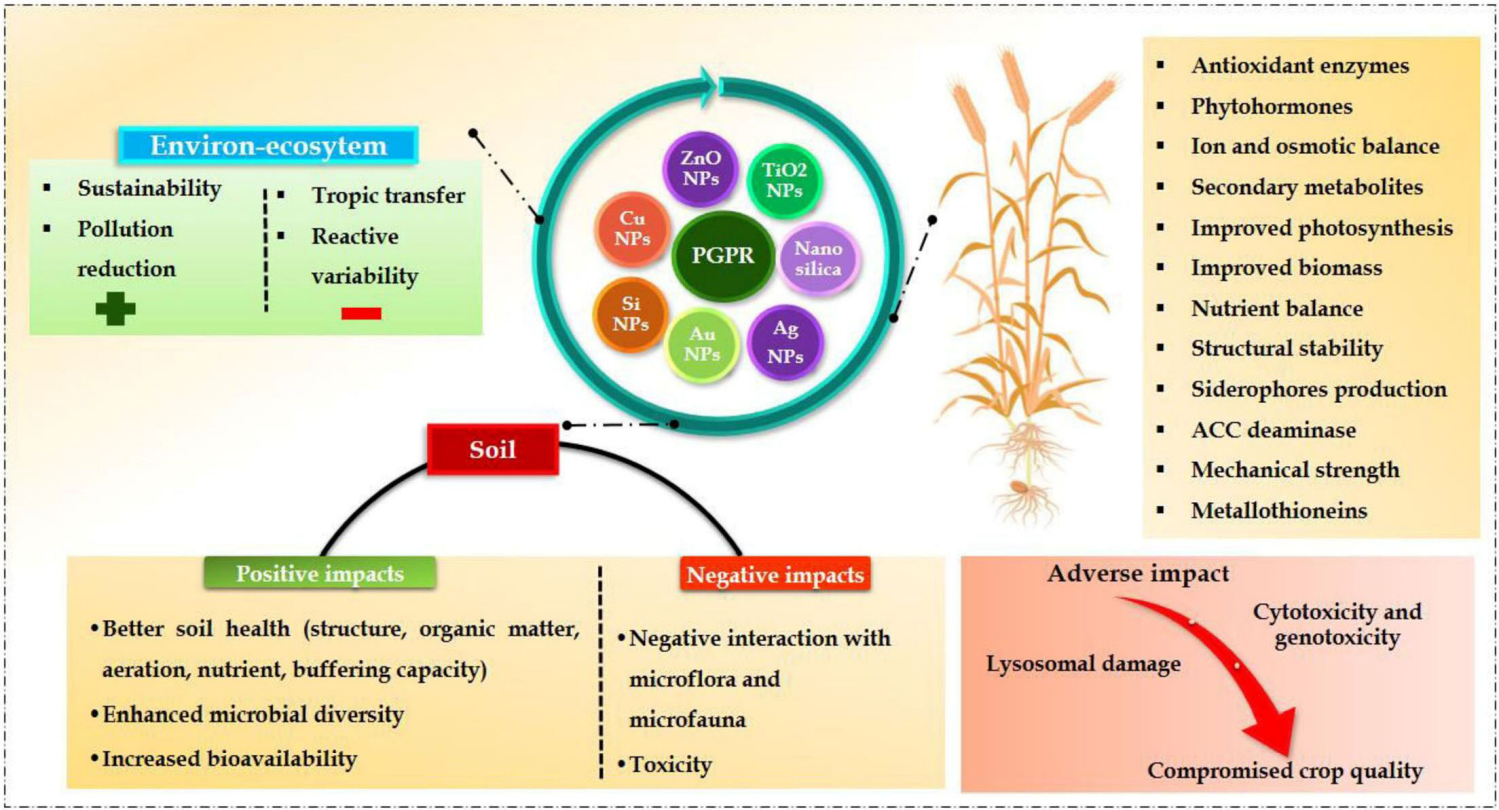
An overview of the interactive effects of NPs and PGPRs on plant, soil and enviro-ecosystems.

Most PGPRs have been reported and confirmed to improve plant productivity by reducing environmental challenges (Singh et al., 2021; Kumari et al., 2023a). Through both direct and indirect strategies, it may enhance the overall quality of soil and plant yield (**Figure 1**) (Sharma et al., 2023; Rajput et al., 2023a). The functions of PGPR occur through direct mechanisms such as nitrogen fixation, phosphate, and potassium solubilization, and the production of growth-promoting phytohormones like indole acetic acid (IAA) and siderophores. However, indirect mechanisms are associated with the production of lytic enzymes and antibiotics, dropping the soil pH and producing exopolysaccharides. Several studies have assessed the efficacy of PGPR for maintaining a sustainable agroecosystem in normal and stressful conditions (Oleńska et al., 2020; Magnabosco et al., 2023; Timofeeva et al., 2023). Numerous articles and meta-analyses have observed the beneficial impacts of NPs on soil health and agronomic productivity as well as the variables that facilitate the ameliorative role of NPs (Urra et al., 2019; Sharma et al., 2020; Verma et al., 2022a, b). The NPs have also alleviated various environmental stresses during plant development (Dilnawaz et al., 2023; Pramanik et al., 2023; Verma et al., 2023a).

Sustainable food security and safety have become extremely challenging in the 21^st^ century, particularly in developing nations with limited resources. The teeming millions in the developing world associated with the era of climate change threaten agricultural crop production and management, a serious challenge to sustainable agriculture (Fanzo et al., 2017; Watts et al., 2018). According to the Food and Agriculture Organization of the United Nations, over 2 billion people do not have enough food to eat due to the COVID-19 pandemic. Agriculture systems and food have already experienced significant modifications, but additional research has to be done in light of the transforming global landscape (Frona and Szenderák 2018; Kakaei et al., 2022). Land reforms, modified water management, stress-tolerant cultivars, increased fertilizer use, better seed, pesticide use, genetically modified crops, plant growth regulators, and soil amendments are some of the approaches used to improve soil quality and crop yields (Urra et al., 2019; Abdul Aziz et al., 2022; Verma et al., 2022c; Akanmu et al., 2023).

**Figure 2:**
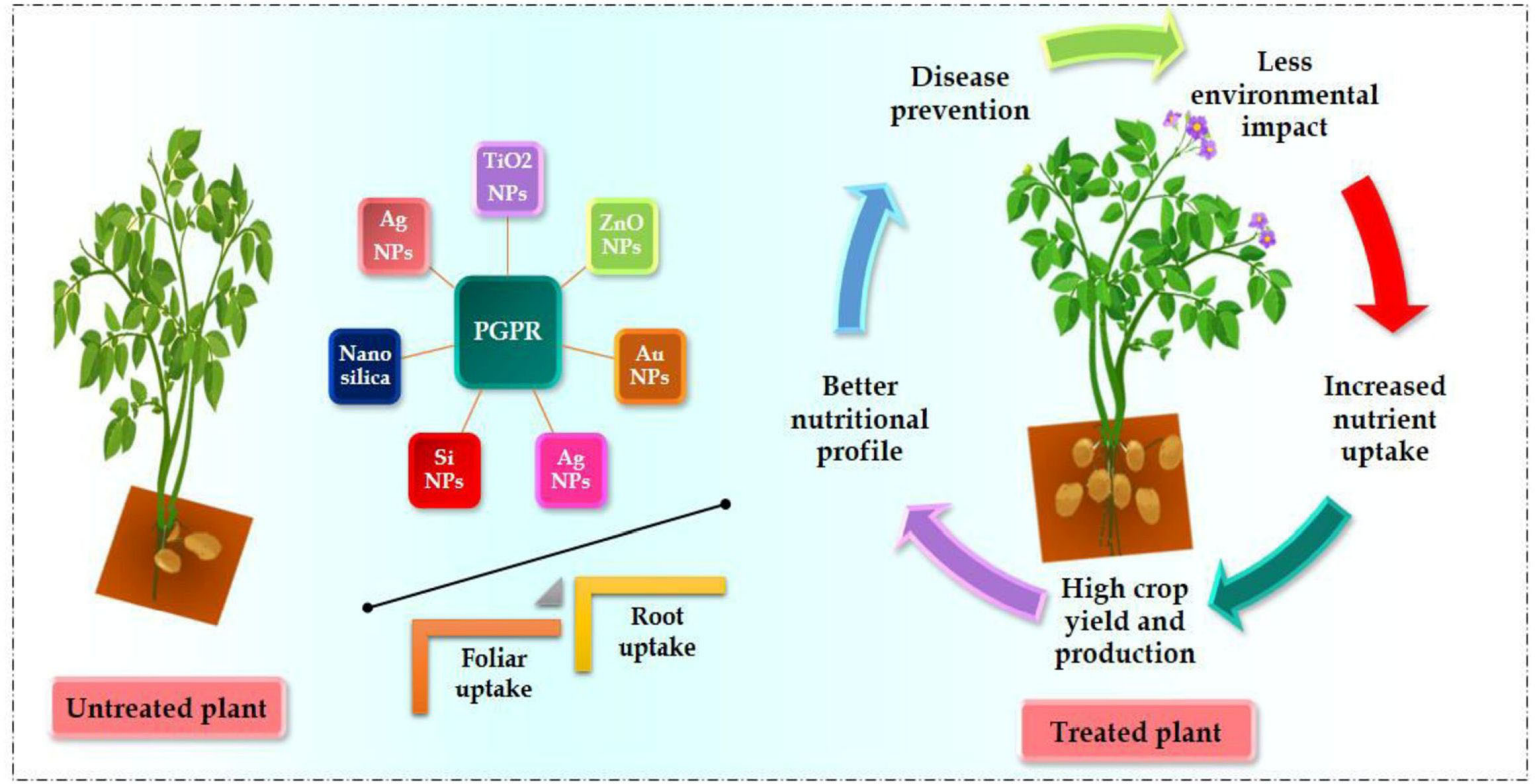
Advantages of NPs and PGPRs applications in food security and safety.

Crop yield downregulated because of various environmental stresses (Verma et al., 2020a; Kumari et al., 2022; Patni et al., 2022; Verma et al., 2023b). Nearly 6% of the world’s total surface area (1125 mha), impacted by salinity; it includes 20% of agricultural land and 33% of irrigated land. The loss of productivity from saline soil can exceed 46 mha annually (Hossain, 2019; Negacz et al., 2022). Annually, 1.5 mha of cropland is lost to saltwater due to soil erosion. Production of crops and animals requires more water as agriculture worldwide, provides 70% of total water returns. Water deficit is a major abiotic stressor prevalent in sub-tropical and tropical regions around the world (Kumari et al., 2022). Furthermore, droughts are becoming more severe due to the era of climate change. Water for agriculture has the potential to become more in requirement globally by 60% upto 2025. The development of plants and production is regularly reduced during drought stress because of insufficient nutrient availability, lower leaf photosynthetic CO_2_ assimilation rate and inadequate water availability (**Figure 2**). Moreover, dehydration increases plants’ biological ethylene synthesis, inhibiting the length and development of their roots (Hanjra and Qureshi, 2010; Mancosu et al., 2015; Boretti and Rosa, 2019; Verma et al., 2023b).

Heavy metals in soils are a major abiotic stressor that reduces agricultural output. Around the world, substantial amounts of heavy metals are frequently present in the soil because of several natural and human activities. Globally, over 10 million locations of polluted soil have been monitored, with a majority of 50% polluted areas with toxic ions. The toxic ions come into the agricultural land from different types of industries, coal burning, wastewater irrigation systems, petrochemical/hydrocarbon spillage, coal combustion, animal waste, and sewage sludge (Boretti and Rosa, 2019; Urra et al., 2019; Rashid et al., 2023; Verma et al., 2023b).

Currently, co-applying PGPRs and NPs has been used in several studies to enhance agronomic productivity and soil quality in different scenarios (**Figures 1**, **2**). The explicit assumption in these studies has been that the NPs would make more nutrients available and generate a conducive habitat for the PGPRs to establish. In response, the latter would carry out their specific work (production of plant hormones, solubilization of nutrients, etc.) at maximum levels. Both stressed and non-stressed soils were used for these analyses. In the meantime, these demonstrations have yet to undergo an in-depth synthesis and critical evaluation. This review article aims to address the knowledge gap. Additionally, highlights the research avenues that will be explored soon to fully utilize the combined potential of PGPR and NPs for sustainable agroecosystems in the years to come.

## Enhancing Soil Quality through Nanoparticle and PGPR Interactions

Because the soils carry out a wide range of ecological functions, it is characterized from the viewpoint of those functions. From the perspectives of concurrent agriculture and the environment, it is described as “the capacity of a soil to function within ecosystem and land-use boundaries to sustain biological productivity, maintain environmental quality, and promote plant and animal health” (Verma et al., 2022d; Anikwe and Ife, 2023; Sharma et al., 2023). The main chemical elements of soil quality are soil organic matter, pH, and accessible macronutrients (nitrogen, phosphorus, and potassium). Comparably, the most widely utilized biological indicators are soil respiration, microbial biomass, nitrogen mineralization, and extracellular enzymatic activities, whereas the most used physical indicators are bulk density, structural stability, and the retention of water (Nielsen and Winding, 2002; Bunemann et al., 2018). This review will evaluate the contribution of co-applying NPs and PGPR to enhance soil quality based on these parameters. Co-applying NPs and various PGPRs has been suggested as an effective approach to improving soil quality. Because NPs provide PGPR with a substrate with a high surface area and increased nutrition for their survival, their presence may enhance PGPR efficiency (Nayana et al., 2020; Akhtar et al., 2021; Alharbi et al., 2023; Rajput et al., 2023b; Verma et al., 2023b). The impact of co-applying different PGPRs and NPs on crop productivity and soil quality has been explored in the subsequent sections (**Figures 1**, **2**; **Table 1**).

**Table 1:** Influence of co-applied PGPRs and nanoparticles on plants in response to normal and stressed conditions.

Stress	Crop	PGPR strains and NPs	Impact on crop and soil properties	Source
Salinity	Wheat (*Triticum aestivum* L.)	*Azospirillum lipoferum* SP2, *Bacillus coagulans* NCAIM B.01123, *Bacillus circulance* NCAIM B.02324, and *Bacillus subtilis* MF497446 with foliar spray of ZnO-NPs (950 g ha^−a^ and 500 mg L^−g^)	The interactive functions of PGPR and ZnO-NPs safe wheat plants during salinity stress via upregulating antioxidative enzymatic responses, such as, CAT, POD, and SOD (47, 102 and 106%), and uptake of K^+^ (27%) as relative to control growing plants. However, alleviation of excess stress via combined application was illustrated by the considerable reduction in membrane stability index (EC), proline, MDA, and H_2_O_2_ content. Enhanced N uptake in treating plants (57%) with application of PGPR+ZnO-NPs. Upregulated the soil urease (80%) and dehydrogenase (232%) activity during saline stress with combined application of PGPR and ZnO-NPs.	Alharbi et al. (2023)
Salinity	Sugar beet (*Beta vulgaris* L.)	*Pseudomonas koreensis* and *Bacillus coagulans* (1x10^8^ CFU/ml) with Si-NPs (12.5 ppm)	The application of PGPR, Si-NPs, and their interaction in upgrading agronomic responses, and productivity of sugar beet exposed to normal and saline water irrigation in salty soil. High saline soil and salty water irrigation enhanced imbalance of ions (K^+^/Na^+^ ratio) and reduced the RWC, relative membrane stability index (RMSI), stomatal conductance, and photosynthetic pigments. The combined application reduced oxidative stress indicators (H_2_O_2_ and MDA) and Na^+^ ions while upregulating the enzymatic activities like SOD (1.9-folds), CAT (1.4-folds), and POD (2.5-folds) as compared normal and excess soil salinity and irrigation of saline water.	Alharbi et al. (2022)
Water deficit andheat	Wheat (*Triticum aestivum* L.)	Single and combined PGPR (*Pseudomonas* sp.) and ZnO-NPs (10 ppm)	Enhanced plant performance, and stress resistance efficiency. Interaction of NPs and *Pseudomonas* sp. protect from stress conditions by producing more proline, SOD, POD, CAT, APX, GR, DHAR and ABA levels. The highest recovery of stress was monitored by the leaf membrane stability reduction, H_2_O_2_ and MDA content. Overall, combined treatment may protect plants mortality from heat and water deficit or both conditions.	Azmat et al. (2022)
Salinity	Onion (*Allium cepa* L.)	*Bacillus pumilus* and *Pseudomonas moraviensis* with Ag-NPs (5ppm)	*Bacillus pumilus* associated with Ag-NPs better performed for the stimulation of plant growth. The higher soil moisture content was observed in saline stressed plants but the inoculated (PGPR) plants and Ag-NPs single and combined with PGPR exhibited loss in the salt induced retention in the moisture of soil. Interaction of Ag-NPs and PGPR and single enhanced the chlorophyll a+b and carotenoids contents during saline stress conditions. The Ag-NPs enhanced the content of sugar and proline.	Jahangir et al. (2020)
Waste/contaminated water irrigation	Maize (*Zea mays* L.)	*Pseudomonas* sp., *Pseudomonas fluorescence* and *Bacillus cereus* with Ag-NPs	The colony forming unit of the PGPR was inhibited by Ag-NPs, but regulated by contaminated water. The Ag-NPs augmented the PGPR induced enhancement in root morphology. The application of Ag-NPs, root-shoot ratio was varied. The enzymatic activities (POD and CAT) were found higher by Ag-NPs and contaminated waste irrigation application. The Ag-NPs regulated phytohormones, such as ABA (~35%), IAA (56%), and GA (83%), enhanced proline level (71%).	Khan and Bano (2016)
No stress	Maize (*Zea mays* L.)	*Bacillus* sp. with Ag-NPs	The significant increment was observed in seed germination (87.5%). The highest plant and root growth was found in applied *Bacillus cereus* with Ag-NPs.	Kumar et al. (2020)
No stress	Cabbage (*Brassica oleracea* L.)	*Nocardiopsis* sp. individually or combined applied with Se-NPs	Enhanced fresh-dry mass and glucosinolate uptake. The myrosinase activity significantly upregulated via sprouts of seeds and consequently increased the amino-acid-derived glucosinolate induction. However, the antibacterial activities were upgraded.	AbdElgawad et al. (2023)
Galaxolide-contaminated soil	Soybean (*Glycine max* L.)	*Actinobacterium* sp. with Se-NPs (25 ppm)	The excess uptake of H_2_O_2_ (+180%), MDA (+163%), and oxidation of protein (+125%), indicating oxidative stress in galaxolide-toxic plants. However, excess uptake of detoxification activity markers, such as phytochelatins (+33%) and metallothioneins (+80%) were observed in mixed applications during contamination of galaxolide. The interactive application of PGPB and Se mitigated the Chl *a* (+58%), gs (+57%) and Fv/Fm (+36%), which resulted in maximum photosynthetic CO_2_ assimilation rate (+36%) and production of biomass (+74%) under galaxolide contamination as relative to normal plants.	Halawani and Aloufi (2023)
No stress	Cucumber (*Cucumis sativus* L.)	*Pseudomonas putida* and *P. stutzeri* (@ 106 cells/ml) with Ag-NPs (5-ppm), foliar spray	Ag-NPs upregulated length of roots but reduced plant length of biomass. Leaf protein, proline, phenolics, flavonoids, Chl b, a+b, sugar and Phenylalanine Ammonia-Lyase (PAL) activities were enhanced as compare to control plants. Ag-NPs also suppressed the PGPR effect for the length of root and shoot but augmented the contents of protein and phenolics. Ag-NPs and PGPR increased flavonoids and PAL, SOD and CAT activities in plant leaves. Ag-NPs enhanced the PAL, CAT and SOD responses in both varieties. The application of *Pseudomonas putida* can be applied either single or in mixed with Ag-NPs to upregulate the antioxidantive and defense enzymatic responses.	Nawaz and Bano (2020)
Excess Fe and Mn	Bitter gourd (Momordicacharantia L.)	*Pseudomonas stutzeri* (10^8^ cells/ml) with Ag-NPs	Carotenoids, protein, and proline activity were found higher as 366, 450, and 678% in bore well water with PGPR and Ag-NPs treatments. *Pseudomonas stutzeri* was more significant than Ag-NPs to minimize oxidative stress with highest carotenoids, flavonoids, proline contents, and enzymatic activities, such as SOD and CAT.	Tariq and Bano (2023)
Chromium	Rice (*Oryza sativa* L.)	Chromium-resistant bacterium *Staphylococcus aureus* and Fe-NPs (20 ppm)	Fe-NPs significantly upgraded plant performance, production, and leaf gas exchange responses by upregulating the Chl content and mitigating the damage of oxidative stress. Chromium-tolerance bacteria (*S. aureus*) increased the significant potential of Fe-NPs by transformation of chromium (Cr^6+^) ion into less toxic form of chromium (Cr^3+^). The bacterial inoculation decreased the accumulation of Cr by plant roots via adsorption of Cr ions.	Alharby and Ali (2022)
Chromium	Sunflower (*Helianthus annuus* L.)	*Staphylococcus aureus* with CeO_2_-NPs (25-50 ppm)	CeO_2_-NPs significantly enhanced plant performance and crop productivity, decreased oxidative stress, and increased antioxidative enzymatic activities during chromium stress condition. *S. aureus* upregulated the potential role of NPs in mitigating metal toxicity. The highest enhancement was observed in applied NPs and *S. aureus*. Increased Chl content and reduced leaf membrane stability index.	Ma et al. (2022)
Cadmium polluted soil	White clover (*Trifolium repens* L.)	*Pseudomonas fluorescens* (10^7^ CFU/kg soil) and TiO_2_ -NPs (100-1000 mg/kg)	Interactive role of TiO_2_-NPs and PGPR upgraded plant development and Chl level as compare to control. Application of TiO_2_ NPs to rhizospheric soil potentially enhanced the uptake efficiency of *T. repens*. TiO_2_ NPs and PGPR can decrease the TiO_2_-NPs for phytoremediation of toxic ions. Combined application maintained *T. repens* growth in polluted soil and increased accumulation of Cd in plants.	Zand et al. (2020)
No stress	Okra (*Abelmoschusesculentus* L.)	*Pseudomonas libanesis* with Se-NPs	Enhanced phytochemicals (25–35%), height of shoot and root (25–35%), and fruit quality with the application of Se-NPs (75 ppm) to avoid the Se-NPs bioaccumulation in the agro-ecosystems.	Sonali et al. (2023)
No stress	Maize (*Zea mays* L.)	*Bacillus megaterium, Bacillus brevis, Pseudomonas fluorescens* and *Azotobacter vinelandii* with Si-NPs	Enhanced the efficiency of seed germination, plant development and productivity	Karunakaran et al. (2013)
No stress	Wheat (*Triticum aestivum* cv. Stava)	*Bacillus thuringiensis* AZP2, *Paenibacillus polymyxa* A26 with TiO_2_-NPs	Upreguleted PGPR activities and their colonization	Timmusk et al. (2018)
No stress	Oilseed rape (*Brassica napus* L.)	*Bacillus amyloliquefaciens* subsp. plantarum UCMB5113 with TiO_2_-NPs	Protected plants from the fungal infection, i.e., *Alternaria brassicae*	Palmqvist et al. (2015)
No stress	Cowpea (*Vigna unguiculata* L.)	P*seudomonas monteilii* with Au-NPs	Increased the production of IAA by *P. monteilii* with Au-NPs (50 µg/mL). Au-NPs upgraded plant agronomic responses	Panichikkal et al. (2019)
No stress	Maize (*Zea mays* L.)	*Bacillus* sp. with nanozeolite (50 ppm)	Agronomic response, such as length of plants, leaf area expansion, leaf numbers, photosynthetic pigments and leaf protein were significantly upregulated. Biochemical activities of soil were significantly enhanced, i.e., dehydrogenase, fluorescein diacetate hydrolysis and alkaline phosphatase activities.	Khati et al. (2018)
No stress	Soybean (*Glycine max* L.)	*Bradyrhizobium japonicum*, *Pseudomonas putida*, *Azospirillum lipoferum* with Zn-NPs (0.3-0.9g/mL^1^	Plant length, number of nodules, grain yield-weight enhanced	Seysdsharifi and Khoramdel (2016)
No stress	Maize (*Zea mays* L.)	*Bacillus* sp. with nanochitosan	Increased the frequency of seed germination (60 - 97%), length of plants (1.5-fold), and leaf area expansion (2-folds). Soil biochemical activities, i.e., dehydrogenase, fluorescein diacetate hydrolysis and alkaline phosphatase were upregulated. Plant metabolites increased, such as alcohols, acid ester and aldehyde compounds.	Khati et al. (2017)

## Nutrient Enhancement in Soil: The Role of Nanoparticles and PGPR

Numerous demonstrations assessed the impact of co-applying PGPR and NPs on soil quality, characterizing the physical, chemical, and biological characteristics of soil (**Table 1**). When NPs and PGPRs are applied together, generally found to enhance the level of mineral nutrient content in rhizospheric soils when compared to a single application of NPs or PGPR (Akhtar et al., 2021; Singh et al., 2021; Alharbi et al., 2023; Tang et al., 2023). The combined use of NPs and PGPRs resulted in increased soil nitrate levels compared to a single dose of nitrogen. However, PGPR and NPs enhanced organic carbon, phosphorus, and nitrogen availability compared to normal plants. Nanoparticles sprayed on plant leaves at low concentrations since they are economical and environmentally beneficial (Ameen et al., 2021; Liu et al., 2022; Verma et al., 2023a). NPs acquired more interest from plant physiologists because of their usage as nano-growth regulators to improve the growth and development of plants, mainly owing to the effective supply and accumulation and translocation of required minerals (Awan et al., 2020; Kupe et al., 2023). When mixed in soil, they dissolve nutrients due to dissolution and decomposition under the influence of soil properties and the activity of microbes. PGPR, especially those that solubilize organic phosphate, accelerates the accumulation of mineral nutrients from NPs. By utilizing PGPR and NPs together, soil microorganisms are spared from the need to seek out certain nutrients, enabling them to focus on the uptake of other essential elements. This shift enhances enzyme activity and facilitates the release of additional nutrients, thus promoting a more efficient nutrient acquisition process and overall soil fertility enhancement (Ameen et al., 2021; Liu et al., 2022; Rajput et al., 2023a).

## Improving soil water retention with nanoparticles and PGPR

With their higher surface area-to-volume ratio, NPs can potentially increase soil water-holding capacity (WHC), especially those with coarse textures. The ameliorative impact of NPs on water retention capacity has also been found in multiple states of the experiments evaluating the co-application of NPs and PGPR. In comparison to PGPR and NPs application only, combined application of PGPR and NPs enhanced soil water holding capacity (WHC) (Nayana et al., 2020; Tang et al., 2023). While single use of PGPR has never been shown to improve soil WHC and water content, it can increase water-deficit resistance capacity to crop plants. However, enhanced WHC by the application of NPs, synergize with PGPR given that the nutrient cycling, breakdown of soil organic matter, and microbial signaling considering higher soil moisture levels [**Figure 2**] (Verma et al., 2020b; Ahmad et al., 2022; Chieb and Gachomo, 2023). It should be highlighted that the indirect significance of NPs with PGPR application has yet to be conducted.

## Modulating soil microbial communities

Nanoparticles enhance various physicochemical characteristics of soil, eventually facilitating the function of indigenous soil microbial populations. NPs can boost WHC, soil pH, substrate, and nutrient availability, enhancing microbial biomass, abundance, and diversification (Rajput et al., 2023a). Furthermore, it has been demonstrated that NPs upregulate the nodulation of the natural rhizobia with legume plants. This is due to the enhancement in aeration provided by NPs that access more air to nodule bacteria, which can persist for a long time on the porous surface of NPs before colonization in the roots (Swarnalakshmi et al., 2020; Flores-Duarte et al., 2022).

The mutualistic relationship between existing microorganisms and plants may be further enhanced by adding NPs (Ameen et al., 2021). The phosphate-solubilizing bacteria *Pseudomonas* sp. increased the availability of phosphorus in soil presumably by solubilizing it from the NPs thereby increasing the colonization of roots and overall plant development (De Souza-Torres et al., 2021). The general abundance of some microbial groups in soil may increase with the interactive role of NPs and PGPR, which improves soil health throughout (**Table 1**). The authors attributed it increase in advantageous bacteria to the improved soil organic matter content and its breakdown due to the interacting effect of NPs and the inoculant (De Souza-Torres et al., 2021; Ahmad et al., 2022; Rajput et al., 2023b).

## Catalyzing soil enzyme activity with nanoparticles and PGPR

Research studies on various intra- and extracellular enzymes have also been used to evaluate the potential benefits of applying NPs and PGPR simultaneously. Soil urease and dehydrogenase enzymes activity was enhanced by applying PGPR and NPs compared to single-use of any of them (Alharbi et al., 2023; Upadhayay et al., 2023). Jabborova et al. (2021) observed that co-inoculation of PGPRs with NPs resulted in higher levels of protease, alkaline, and acid phosphomonoesterase than a single application of PGPRs. Interactive application of *Bacillus subtilis* with NPs was significantly observed in the activities of invertase and catalase in soil than in single use of NPs (Medina-Velo et al., 2017; Khanna et al., 2021). Ultimately, it has been revealed that the PGPR with NPs increases the enzymatic activity of soil phosphomonoesterase in acidic and alkaline conditions, as well as that of sucrase, urease, protease, and invertase. By defining the direction and intensity of nutrient transformation processes in soil, these enzymes can improve soil fertility by stimulating biochemical processes within the ecosystem. There is a direct correlation between soil nutrients and the enhanced enzyme activity caused by PGPR and NPs (Mushtaq et al., 2020; Ahmad et al., 2022; Rajput et al., 2023b). Research is still lacking on how co-application of NPs and PGPRs affects leucine aminopeptidase and N-acetyl-glucosaminidase, significant N-cycling enzymes. These enzymes catalyze the complex proteinaceous compounds in soil. The amount of mineral nitrogen added to the NPs can be determined by measuring the activity of these enzymes in the presence of PGPR since the NPs are organic materials that contain organic proteins (**Figures 1**, **2**; **Table 2**).

**Table 2:** Some of the stress-responsive genes in plants that can be regulated by NPs and PGPRs.

Gene Category	Functions	Examples of Genes/Proteins	Source
Antioxidant Genes	Neutralize reactive oxygen species (ROS)	Superoxide dismutase (SOD), Catalase (CAT), Peroxidases (POD), Ascorbate peroxidase (APX)	Akhtar et al., 2021; Ali et al., 2021; Azmat et al., 2022; Sun et al., 2022
Heat Shock Proteins (HSPs)	Protect plants against high temperature and other stressors	*HSP70, HSP90*	Zhao et al., 2012
Water Stress Genes	Regulate water flow in cells	Aquaporins, Dehydrins, *P5CS, CAT1, DREB2*, dehydration-responsive element-binding proteins, *“HsfA1a,” “SlAREB1,” “LeNCED1,” and “LePIP1”*	Mahakham et al., 2017; Raeisi Sadati et al., 2022; Subotic et al., 2022; Mohamed and Abdel-Hakeem, 2023
Salinity Stress Genes	Maintain ion homeostasis and salt tolerance	Pathway of Salt Overly Sensitive (SOS) genes, auxin responsive proteins (ARP), cAPX, DREB, MnSOD, and GST genes	Hezaveh et al., 2019; Moradi et al., 2022
Ethylene Responsive Factors (ERFs) and biosynthesis genes	Regulate plant responses to adverse agroclimatic conditions	*ERF1, ERF5, ACC deaminase*	Fadiji et al., 2022
Pathogenesis-Related (PR) Proteins	Involved in defense against pathogens and stress responses	*PPO, PR1, PR5*, β-1,3-glucanase	Elmer et al., 2018
ABA-Related Genes	Regulate responses to abiotic stress via the ABA hormone	ABA insensitive (*ABI*) genes, *PYR/PYL* receptors	Azmat et al., 2022
Salicylic Acid (SA) responsive and Pathway Genes	Defense responses modulation	SA responsive PR (pathogenicity related proteins) genes (PR1 and PR2)	Cai et al., 2020
Nitrogen Assimilation Genes	Uptake and metabolism of nitrogen	Nitrate reductase (NR), Glutamine synthetase (GS), nitrification related *amoA1 and amoC2* genes	Yang et al., 2013
Phytochelatin Synthase	Chelation of heavy metals for tolerance	PCS (Phytochelatin Synthase) genes, siderophores	Zand et al., 2020
Glutathione S-Transferases (GSTs)	Detoxification processes within plant cells	GST family genes	Moradi et al., 2022

It provides an overview of various gene categories that may be impacted by the presence of NPs and PGPRs in plants, along with their general functions in stress response.

## Nanoparticles and PGPR: a synergistic approach to combat environmental stresses

Crops that grow with low pesticide application concentrations, higher nutritional values, and disease resistance are necessary for sustainable agriculture. In recent decades, the widespread application of expensive agrochemicals in agriculture has prompted the development of more environmentally friendly substitutes, like PGPR and NPs (Medina-Velo et al., 2017; Ahmad et al., 2022). PGPR and NPs have been broadly mentioned for their significant impacts on plants. However, utilizing PGPR and NPs together has been more successful in plant production in recent years than using PGPR or NPs individually. Multiple research projects have documented the significant role of PGPR and NPs in enhancing crop productivity (Mushtaq et al., 2020; Khanna et al., 2021; Rajput et al., 2023a). Similarly, the combined use of *Alcaligenes* sp. with NPs promoted fresh and dry mass, plant length, yield, and quality of fruits over a single use of PGPR. PGPRs with NPs have also been evaluated during decreased fertilizer frequency to reduce the greenhouse gas emissions associated with the fabrication of ammoniac fertilizers and their volatilization (Awan et al., 2020; Mushtaq et al., 2020; Khanna et al., 2021; Shah et al., 2021).

Application of *Enterobacter, Pseudomonas, Azospirillum, Agrobacterium* and NPs enhanced sustainable agriculture production. The interactive use of PGPR and NPs may enhance seed germination frequency, height of plants, dry-fresh biomass, and crop productivity than the single use of PGPR or NPs (Medina-Velo et al., 2017; Fadiji et al., 2022; Sharma et al., 2023). This combination can work in different directions. In the direct mechanisms, the usual generation of plant hormones by the PGPR, such as indole acetic acid, siderophores, etc., and enhancing soil minerals’ availability through phosphate solubilization and N_2_ fixation contributes to better plant performance and productivity. The existence of NPs can assist the withstanding of the PGPR in larger numbers in addition to providing nutrient-rich substrate thereby leading to improved efficiency by the PGPR ultimately boosting the production of plants (Mushtaq et al., 2020; Khanna et al., 2021; Ahmad et al., 2022; Rani et al., 2023).

It has been widely recognized that the PGPR reducing a wide range of environmental stresses that impede the growth and development of plants. Many researchers have demonstrated their effectiveness in combating drought, soil flooding, salinity, low and high light intensities, nutritional imbalance, and heavy metal contamination. Plants can develop stress tolerance efficiency by exploiting the ability of PGPR to release exopolysaccharides in dry environments (Vurukonda et al., 2016; Ahmad et al., 2022; Chieb and Gachomo, 2023). In saline environments, it increases water absorption, decreases stomatal conductance, boosts potassium accumulation at the cost of sodium, reduces the direct negative impacts of soil salinity, and increases antioxidant enzyme activities. All these alterations assist in the improved growth of plants in saline environments. Similarly, it has been observed that the PGPR improves overall nutrient uptake while also immobilizing and reducing the uptake of heavy metals by plants, thereby reducing the toxicity caused by heavy metals (Bishnoi, 2014; Islam et al., 2014; Kaushal and Wani, 2016; Kumar et al., 2017; Etesami and Maheshwari, 2018). *Bacillus pumilus* in linked with Ag-NPs performed better for the stimulation of onion plant growth. Combined application exhibited a reduction in the salt-induced retention in the moisture of rhizospheric soil and enhanced protein content of bulb, reduced leaf flavonoids. Ag-NPs enhanced sugar and proline levels. *Bacillus pumilus* proved to be more significant during control conditions to all growth agronomic responses but *Pseudomonas moraviensis* potentially coped in response to saline conditions (Jahangir et al., 2020).

Numerous articles have reviewed these findings discussed in the **Table 1**. Additionally, it has been demonstrated that NPs improve salt tolerance, reduce drought stress, and minimize the toxicity that organic and inorganic soil contaminants cause in plants (Rasheed et al., 2022). Drought stress benefit in NPs-amended soils occurs through higher water holding capacity to large surface area-to-volume ratio of NPs (El-Saadony et al., 2022; Verma et al., 2022b, c; Verma et al., 2023a). Similarly, plants in soil amended with NPs mitigate soil saline conditions due to reduced osmotic stress to increased soil water content and decreased Na^+^ absorption caused by Na^+^ transient binding on sorption sites on NPs. The primary approach by which NPs reduce the toxicity stress of organic and inorganic heavy metals sorption. Multiple studies have reviewed all these applications of NPs against diverse environmental stressors (Azameti and Imoro, 2023; Faizan et al., 2023; Rajput et al., 2023b). Recently, some studies have explored the possibility of combining PGPR and NPs to mitigate the environmental circumstances for plant development with the assumption that both additives would act synergistically (**Table 1**). Synergies between PGPR and NPs have been actively explored in these studies.

## Soil quality enhancement: the combined power of PGPR and nanoparticles

Improved soil quality occurs from the combined application of PGPR and NPs, which perform multiple functions to mitigate the effects of drought stress (**Table 1**). When the soil water content was half the field capacity (50% of soil moisture level), applying NPs and PGPR jointly substantially improved the pH, EC, nitrate, phosphorus, extractable K, and organic matter when compared by applying NPs and PGPR separately [**Figures 1**, **2**] (Akhtar et al., 2021; Rajput et al., 2023a). Sun et al. (2022) reported that when the FeO-NPs were treated with arsenic (As)-contaminated soil with the PGPR *P. vermicola*, the outcomes were notably enhanced, yielding more effective results. Consequently, results demonstrated that the *P. vermicola* and FeO-NPs applied combined may mitigate As (arsenic) toxicity in seedlings of *Trachyspermum ammi*, improving plant growth and composition under metal stress observed by balanced exudation of organic acids (Sun et al., 2022). The developing direction from this research suggests that the higher water holding capacity and concurrent reduction in drought stress strengthen the survival and abundance of the PGPR, which perform their activities better (Ahmad et al., 2022).

In terms of soil quality, salt influences the composition of the microbial community in the soil and reduces biomass and microbial activity. Furthermore, under saline conditions, Na^+^ exceeds K^+^ transport channels, resulting in decreased and inhibited development of plants (Al-Turki et al., 2023). Despite this, it has been demonstrated that co-application of PGPR and NPs in salty conditions induces salt tolerance and plant development, mostly through lowering Na^+^ absorption and elevating the K^+^/Na^+^ ratio. Using two endophytic bacteria, such as *Burkholderia phytofirmans* or *Enterobacter* sp., and NPs drastically reduced saline stress in crop plants by minimizing the uptake of xylem Na^+^ content. On the contrary, combined application greatly enhanced the K^+^ and K^+^/Na^+^ ratio, reducing plant saline stress (Kumawat et al., 2022; Al-Turki et al., 2023; Rajput et al., 2023b). In the same demonstration, the sodium adsorption ratio and Na^+^ in soil solution were reduced by the latter application to adsorption sites and desorption of K^+^ by interactive combination. When inoculated with NPs and *Paraburkholderia phytofirmans*, which can produce exopolysaccharides—significantly reduced the level of Na^+^ in the soil solution, alleviating plant salinity stress (Fu and Yan, 2023).

Combined PGPRs and NPs have synergistic benefits on soil quality because they reduce the Na^+^ level and increase colonization efficiency. PGPR strains co-applied with NPs in salty soil showed higher colonizing efficacy than PGPRs without NPs in the soil (Kumawat et al., 2022; Rajput et al., 2023b). *Enterobacter* sp. with 5% NPs demonstrated increased colonizing efficiency in saline soil than *Burkholderia phytofirmans* with and without NPs. Compared to PGPR inoculation individually; co-applicating an endophytic PGPR with NPs produced in salty soil caused an increase in PGPR colonization in the rhizosphere, root, and shoot interior bacterial population of about 150–250%. In rhizospheric soil, NPs demonstrated downregulation in the Na^+^/K^+^ ratio and improved PGPR root colonization efficiency, reducing soil salinity stress. By releasing mineral elements like K^+^, Ca^2+^, and Mg^2+^ from the soil solution, NPs, and PGPRs maintain the nutritional balance by lowering the concentration of Na^+^ in the soil. As a result, the soil K^+^/Na^+^ ratio gradually improved. Na^+^ in soil binds by exopolysaccharides formed by PGPRs under stress (deMoraes et al., 2021; Kumawat et al., 2022; Fu and Yan, 2023). The specific genes responsive to stress in plants under the influence of NPs and PGPRs can widely depend on the type of stress, plant varieties/species, type of NPs, type of application, and applied PGPRs strains. Some of the stress-responsive genes in plants can be regulated by NPs and PGPRs which are shown in **Table 2**. It provides an overview of various gene categories that may be impacted by the presence of NPs and PGPRs in plants, along with their general functions in stress response.

In polluted soils, the application of PGPR together with NPs has also been explored (**Table 1**). Based on the findings; it is an effective approach for lowering soil contamination from heavy metals. *Enterobacter* sp. microbe with NPs could efficiently expedite the restoration of soil contaminated with cadmium (Cd) toxicity. The combined application of *Bacillus* sp. and NPs enhanced soil enzyme (dehydrogenase) more than NPs, leading to improved biological remediation. This combination also reduced HOAc-extractable Cd levels than independent applications of NPs and PGPR. By lowering the heavy metal’s availability, *Bacillus* sp. applied with NPs significantly reduced the detrimental impact of chromium and improved plant health. PGPRs and NPs immobilize metals via metal-immobilizing bacteria, adsorption, co-precipitation, and complexation, therefore restricting their availability in soil for uptake and translocation (Mitra et al., 2018; deMoraes et al., 2021; Zulfiqar et al., 2023).

## Synergistic role of PGPR and nanoparticles enhancing physiological and yield characteristics

Several studies have demonstrated that the role of PGPR and NPs on plant performance in adverse agroclimatic conditions (**Table 1**). The impact of applying PGPR and NPs together on plant development and productivity has been reviewed, and different physiological and biochemical responses have been triggered (Rajput et al., 2023a). However, a consequence of drought stress is increased plant ethylene levels. It has been demonstrated that the use of ACC-producing deaminase-producing PGPR with NPs may reduce the higher ethylene level in plants caused by drought because the latter increases colonization in the plant rhizosphere and promotes the inoculant survival rate (deMoraes et al., 2021; Al-Turki et al., 2023). In terms of comparison, this resulted in higher plant yields than using PGPR or NPs alone. Comparably, using *P. aeruginosa* and NPs jointly decreased electrolyte leakage substantially compared to applying independently. Also, co-application improved fresh and dry leaf-shoot-root weight compared to a single usage of NPs or PGPR, according to deMoraes et al. (2021). When combined with NPs, several additional PGPRs that produce ACC deaminase, such as *Agrobacterium fabrum* and *Bacillus amyloliquefaciens* have also been shown to increase wheat productivity during severe water stress conditions [**Table 1**] (Al-Turki et al., 2023; Rajput et al., 2023a).

PGPR applied along with NPs to stressed plants, enhanced relative water content, stomatal conductance, Ca^2+^ and K^+^ levels, and reduced proline level. Additionally, researchers observed the reduced electrolyte leakage assisted plants in adapting to drought stress conditions. Drought causes plants to release more ethylene and electrolytes, which inhibits plant growth. By reducing ethylene concentration and electrolyte leakage in plants, co-application of PGPR with NPs may mitigate drought stress in plants. According to experiments carried out by Ahluwalia et al. (2021); Alharbi et al. (2023) and Chandrashekar et al. (2023), PGPR with NPs increased the relative water content, stomatal conductance, chlorophyll, and carotenoids in plants.

The salty soil influences plant development, growth, photosynthesis, lipid metabolism, and protein synthesis (**Figures 1**, **2**). Hormonal imbalances and osmotic changes harm plant growth in saline soils (Singh et al., 2013). It also results in specific toxicity of ions and malnutrition. A different reason is that both sodium and chloride ions restrict plant growth. In certain plants, only chloride ions accumulate in the shoot, while sodium ions are retained in the roots and stems (Kumawat et al., 2022; Fu and Yan, 2023). When PGPRs and NPs are applied in common, their combined effects normally have a more beneficial impact on plant productivity and the elimination of salt stress than separate applications. Under salinity stress, combining NPs and a siderophore-producing strain of *Burkholderia phytofirmans* enhanced the plant height, grain yield, photosynthetic leaf gas exchange, and root and shoot dry weight, respectively. The synergistic ability to integrate PGPR and NPs to ameliorate soil salinity stress for plants has also been confirmed by evidence from multi-year field research experiments (deMoraes et al., 2021; Kumawat et al., 2022; Alharbi et al., 2023). PGPR and NPs play a major role in restoring toxic ions in plants. They can alter, accumulate or eliminate heavy metals (Gulzar and Mazumder, 2022; Wang et al., 2022; Rai et al., 2023).

When combined with NPs, *Enterobacter* sp. significantly facilitated the development of *Brassica napus* in cadmium-contaminated soil (Saeed et al., 2019). Compared to soil without PGPR and NPs under stress conditions, the co-application greatly enhanced shoot and root length, respectively. In addition, PGPR with NPs application reduced Cd uptake in root and shoot under Cd stress conditions relative to individual use of PGPR and NPs, respectively. An increment was observed in ryegrass biomass than the application of NPs, and minimum Cd level was noticed in the interactive application as relative to NPs, PGPR, and control (Jin et al., 2016; Rizwan et al., 2021).

Recent studies performed by Timmusk et al. (2018) underscore the transformative potential of integrating titania nanoparticles (TNs) with Plant Growth-Promoting Rhizobacteria (PGPR) in agriculture. This novel approach has demonstrated a significant boost in plant biomass, particularly under challenging conditions such as drought, salt, and pathogen stress. The synergy between TNs and PGPR not only fortifies plant resilience but also paves the way for sustainable yield improvements. By harnessing the combined power of these two agents, we can unlock new pathways to bolster plant health and productivity, marking a significant step forward in our quest for sustainable agricultural practices.

## Deciphering the interactions: how PGPR and nanoparticles influence plant biology

When NPs are applied with PGPR strains, they provide a habitat for PGPR (i.e., colonization, reproduction, and growth) due to its porous structure, more surface area, and the capacity to absorb microorganisms and organic compounds. Several studies mentioned in **Table 1** demonstrated that the adding NPs to soils increases the growth and abundance of PGPR inoculants. NPs also protected them from other dangerous bacterial infections. It provides energy and the essential nutritional building blocks for inoculants’ survival and growth. Furthermore, using NPs influences the physicochemical characteristics of soils and may increase soil microbial biomass and enzymatic activity. NPs are rich in different nutritional compositions, such as nitrogen, phosphorus, potassium, calcium, magnesium, zinc, etc., depending on the type and frequency of application (deMoraes et al., 2021; Singh et al., 2021; Alharbi et al., 2023; Al-Turki et al., 2023; Fu and Yan, 2023; Verma et al., 2023a).

PGPRs enhance plant growth through direct and indirect processes during normal and stressful situations. Like NPs, the PGPR can either bring in a nutrient from outside via their direct functions such as nitrogen fixation (by nitrogen-fixing bacteria) or solubilize the immobilized nutrients (by phosphate-solubilizing bacteria) thereby contributing to plant nutrition [**Figures 1**, **2**, **Table 1**] (deMoraes et al., 2021). In addition, due to their nitrogen-fixing characteristics, nitrogen-fixing PGPRs like *Paenibacillus polymyxa, Rahnella* sp., and *Serratia* sp. can increase the level of mineral nitrogen in soil solution and restrict it from leaching into the soil (Weselowski et al., 2016; Liu et al., 2019; Ribeiro et al., 2020). A wide range of phosphate-solubilizing PGPRs has been demonstrated to solubilize and provide phosphate in soil for plant uptake (Walpola and Yoon, 2013; Alori et al., 2017; Prabhu et al., 2018; Prakash and Arora, 2019; Gupta et al., 2021; Koczorski et al., 2023; Suleimanova et al., 2023).

The direct phosphorus accumulation from NPs using PGPR has yet to be demonstrated. Similar mechanisms of higher availability of potassium may be predicted because PGPR is known for reducing the soil pH and making the soil potassium available to plants and NPs to be rich in essential minerals (deMoraes et al., 2021; Koczorski et al., 2023). Another direct pathway of the production of ACC deaminase reduces the generation of ethylene-enhanced levels obtained during stress conditions via its breakdown into ammonia and alpha ketobutyrate. *Enterobacter* sp., *Alcaligenes* sp., *Pseudomonas fluorescens, Serratia odorifera, Leclerciaade carboxylata, Agrobacterium fabrum, Bacillus amyloliquefaciens, Pseudomonas aeruginosa*, etc., can be produced by ACC deaminase. These strains have beneficial synergistic effects with NPs during environmental stress mitigation. PGPRs by their indirect processes, i.e., pH regulations, production of exopolysaccharides, and defense against biotic stresses are also associated with plant growth enhancement (Alori et al., 2017; Prakash and Arora, 2019; Singh et al., 2021; Kumawat et al., 2022; Alharbi et al., 2023; Suleimanova et al., 2023).

## Potential drawbacks and risks associated with their use of NPs and PGPRs for sustainable crop development

Nanoparticles offer transformative potential for agriculture through their unique properties, such as high reactivity and the ability to be engineered for specific tasks. Still, they also present environmental and health risks. Concerns include the toxicity of nanoparticles to non-target organisms like soil microbes, plants, and animals, with studies indicating that certain nanoparticles can disrupt soil microbial communities and accumulate in the food chain. Their small size facilitates mobility, raising the risk of environmental contamination and unknown long-term effects (Tourinho et al., 2012; (Dimkpa et al., 2013; deMoraes et al., 2021). Human health concerns also arise from the potential for nanoparticles to enter the body through food consumption, with evidence of their ability to cross biological barriers. Regulatory challenges are significant, as existing frameworks may not adequately address the risks associated with nanomaterials. Despite these risks, the benefits of nanoparticle use in agriculture are considerable, necessitating comprehensive risk assessments, safe handling practices, and clear regulatory guidelines to ensure their safe and sustainable application ( (Grieger et al., 2012; Shah et al., 2021). To ensure a sustainable approach, it is crucial to conduct comprehensive risk assessments, invest in research for understanding long-term effects, and develop nanomaterials that are biodegradable and safe for the environment and human health. Furthermore, establishing clear regulatory guidelines and promoting safe handling practices are essential for mitigating risks (Grieger et al., 2012). By prioritizing safety and sustainability, the agricultural sector can leverage nanotechnology to address global food security challenges while protecting environmental health and biodiversity.

## Conclusions and future perspectives

During adverse agroclimatic conditions, reduced plant growth and crop mortality are prevalent through significant food and commercial cash crops. Given the significant potential of PGPR and NPs to enhance crop productivity and soil health under both normal and adverse conditions, our conclusion highlights the necessity of integrating these technologies into sustainable agricultural practices. The synergy between PGPR and NPs not only offers a path to reducing synthetic fertilizer dependency but also promises resilience against climatic stresses by improving plant performance, production, and fruit or grain quality, as well as soil profile. However, as highlighted in this article, limited field demonstrations have been conducted to assess the significance of the PGPR and NPs’ interactive role in sustainable agriculture. Mechanistic research on the interaction between PGPR and NPs requires more research. An extremely efficient approach to evaluate the combined impact of PGPR and NPs could be long-duration field demonstrations. The potential of the PGPR and NPs for sustainable food production has been independently assessed in reasonably long-duration field studies.

Future research is poised to delve deeper into the mechanistic underpinnings of PGPR and NP interactions, with a strong emphasis on long-term field trials. These studies are critical for understanding the dynamic interactions in various soil types, especially those lacking in organic matter in tropical and subtropical regions. The aim is to develop tailored application strategies that leverage the unique benefits of PGPR and NPs, thereby reducing reliance on synthetic fertilizers and enhancing the environmental sustainability of agricultural systems. This detailed exploration and application of PGPR and NPs hold the promise of revolutionizing farming practices to meet the global food demand sustainably.

## Author contributions

KV: Writing – original draft, Resources, Methodology, Formal analysis, Data curation, Conceptualization. AJ: Writing – original draft, Software, Resources, Formal analysis, Data curation. X-PS: Writing – review & editing, Visualization, Validation, Supervision, Project administration, Methodology, Investigation, Funding acquisition, Conceptualization. SS: Writing – review & editing, Software, Resources, Data curation. AK: Writing – review & editing, Software, Resources, Formal analysis, Data curation. JA: Writing – review & editing, Software, Resources, Formal analysis, Data curation. SKS: Writing – review & editing, Software, Resources, Formal analysis, Data curation. MS: Writing – review & editing, Software, Resources, Formal analysis, Data curation. CS: Writing – review & editing, Software, Resources, Formal analysis, Data curation. Y-RL: Writing – review & editing, Visualization, Validation, Supervision, Software, Project administration, Methodology, Investigation, Funding acquisition, Conceptualization.
